# Arsenolipids in oil from blue whiting *Micromesistius poutassou* – evidence for arsenic-containing esters

**DOI:** 10.1038/srep07492

**Published:** 2014-12-15

**Authors:** Mojtaba S. Taleshi, Georg Raber, John S. Edmonds, Kenneth B. Jensen, Kevin A. Francesconi

**Affiliations:** 1 Institute of Chemistry-Analytical Chemistry, University of Graz, Universitaetsplatz 1, 8010 Graz, Austria; 2 Department of Marine Chemistry, Faculty of Marine Science, University of Mazandaran, Babolsar, Iran

## Abstract

Arsenic-containing lipids in the oil from the blue whiting fish (*Micromesistius poutassou*) were separated into three broad polarity groups and investigated by HPLC and mass spectrometry. A total of 11 arsenolipids including 4 new compounds were identified. The polar lipid fraction constituting 24% of the total arsenolipid content (which totalled 2.16 μg As/g) contained four known dimethylarsinoyl fatty acids and three known dimethylarsinoyl hydrocarbons. The less polar fraction (ca 30% of the total arsenolipids) contained four new dimethylarsinoyl hydrocarbons with chain lengths 22–30 carbons, in addition to more complex arsenicals that hydrolysed to known dimethylarsinoyl fatty acids suggesting they were conjugated carboxylic acids, presumably esters. The rest of the lipid-soluble arsenic (ca 45% of the total) remained in the non-polar fraction together with the bulk of the fish oil lipids, a complex mixture of compounds that precluded identification of the small amounts of arsenolipids.

Although recent results^1^ in the field of biological arsenic chemistry have attracted some heated debate and refutation^2^, the amazing array of organoarsenic compounds produced by organisms continues to attract interest and encourage thoughts of arsenic's serving a possible biochemical role. The newest additions to the range of arsenic natural products are the arsenic-containing fatty acids and hydrocarbons, so-called arsenolipids, which are found at significant concentrations in fish oils^3^,^4^,^5^, in the muscle tissue of fatty fish such as sashimi tuna^6^, herring^7^, and several other species^8^, and in algae^9^,^10^. Another type of arsenolipid, arsenosugar phospholipids (or arseno-glycophospholipids), appears to be prominent in algae^9^,^10^, but those lipids have not yet been identified in fish oils.

The arsenolipids of fish oils appear to fall into three broad polarity groups (polar, less-polar, and non-polar – see Supplementary Fig. S1), as shown by their solvent partitioning properties and their chromatographic behaviours on reversed-phase HPLC. The dimethylarsinoyl group [Me_2_As(O)-] imparts to the arsenolipids considerable polarity, which is moderated by increasing carbon chain length and augmented by functional groups or the degree of unsaturation. A recent study^11^ has shown that arsenic-hydrocarbons are cytotoxic and suggests that the bioaccessability and degree of toxicity of these arsenic-lipids might be related to their polarity and their ability to cross the cell membrane.

Most work on natural lipid arsenicals has focussed on the polar lipids, resulting in the identification of arsenic-containing fatty acids and arsenic-hydrocarbons of mid-length carbon chains. Further insights into the biological chemistry of arsenic might be gleaned from investigations of other lipid fractions. Our preliminary examination of the arsenic compounds in the oil from the blue whiting, a teleost fish, showed that it contained a large amount of the less-polar lipids. We report an investigation using HPLC/mass spectrometry and accurate mass spectrometry of the arsenolipids of blue whiting oil, with the focus on the less-polar lipid fraction.

## Results

### Partitioning of arsenolipids from blue whiting oil

The oil from blue whiting contained 2.16 μg As g^−1^. When the oil was partitioned between hexane and aqueous methanol, 24% of the total arsenic, but only 0.5% of the total mass, was found in the polar aqueous methanol fraction. The hexane layer was further sequentially extracted with aqueous methanol and aqueous ethanol; these extracts contained the same arsenicals (by HPLC/MS) as found in the first aq. methanol extract, but at lower concentrations, and hence the fractions were not investigated further. An aqueous isopropanol extraction of the hexane layer yielded a less-polar group of arsenolipids (ca 30% of the total arsenic), whereas the arsenolipids remaining in the hexane were designated as being non-polar lipids. A flow diagram of the partitioning procedure is shown in Supplementary Figure S2.

### The polar lipid fraction: Identification of seven known arsenolipids

**Figure 1:**
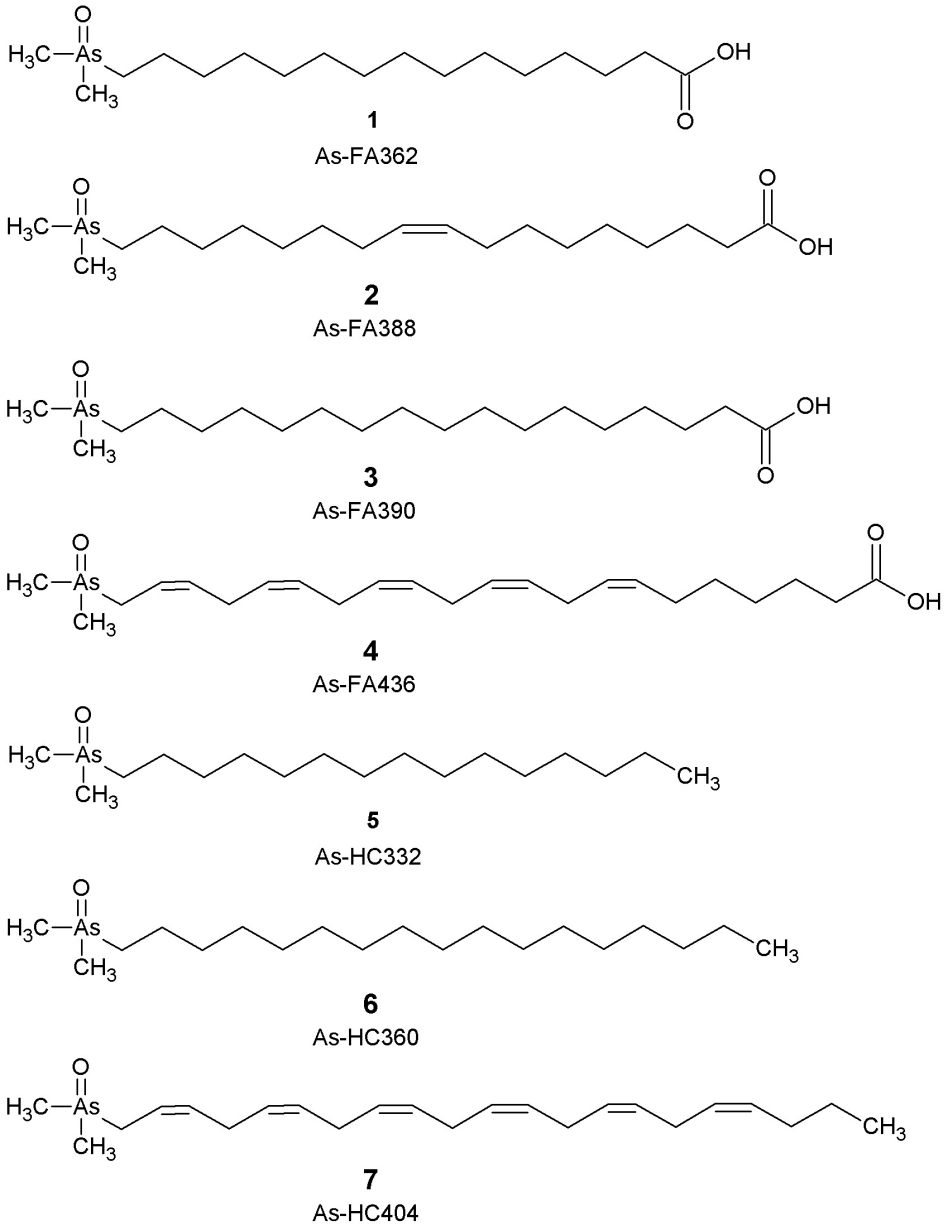
Known arsenolipids found in blue whiting oil. For ease of reference, the arsenic fatty acids and arsenic hydrocarbons are referred to by the abbreviation As-FA and As-HC, respectively, followed by their nominal molecular mass.

The aqueous methanol extract was fractionated by preparative anion-exchange chromatography into an acidic fraction and a non-acidic fraction, which were further investigated by using HPLC/electrospray ionisation-MS, in simultaneous elemental and molecular mode. This method records, at the same time, a signal for arsenic and for selected molecular masses for the HPLC peaks, thereby allowing the assignment of a molecular mass to arsenic-containing compounds^12^. The acidic fraction yielded four known arsenic fatty acids with molecular masses 362, 388, 436 and 390, identified by comparison with arsenolipids previously identified in cod-liver oil^3^ (Fig. 1, compounds **1–4**). These four arsenic fatty acids were estimated to constitute less than 5% of the total arsenolipid content in blue-whiting oil. The non-acidic portion of the polar lipid fraction was similarly analysed by HPLC/ESI-MS and shown to contain three known arsenic-containing hydrocarbons (Fig. 1, compounds **5–7**), first identified in capelin oil^4^.

### The less-polar lipid fraction: (1) Identification of four novel arsenic-containing long-chain hydrocarbons

A portion of the isopropanol fraction was purified by passage through a silica column and elution with methanol. HPLC/ESI-MS in simultaneous arsenic-selective and molecular mass mode revealed several arsenic-containing peaks for which clear protonated molecular masses could be determined showing that the molecular masses were 440, 442, 444, and 542 (Supplementary material Fig. S3). When these compounds were treated with H_2_S their masses increased by 16 mass units, a result consistent with the presence of the Me_2_As(O)- group and its ready conversion to the thio analogue [Me_2_As(S)-]^13^.

**Figure 2:**
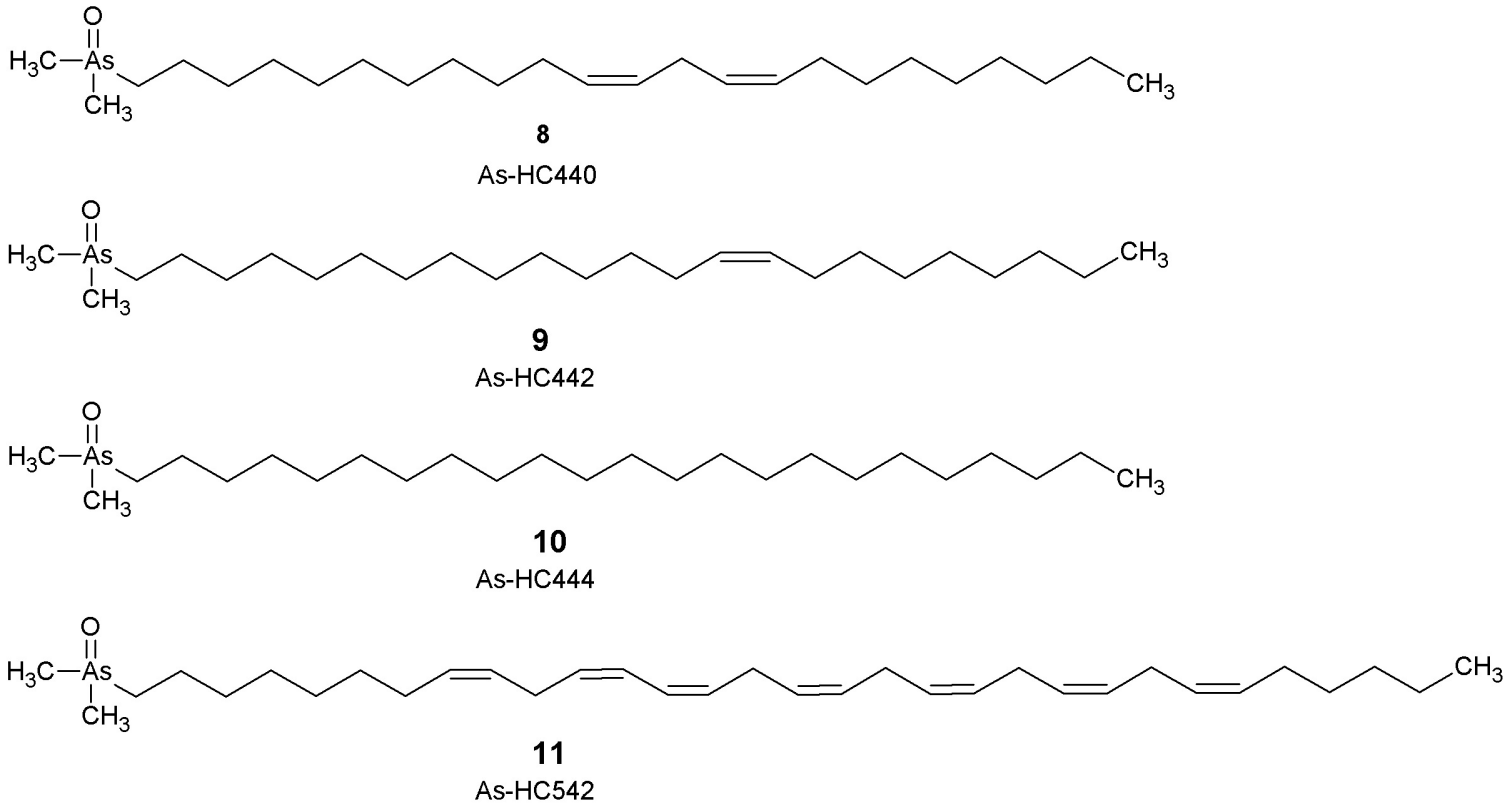
Four new arsenolipids found in blue whiting oil. These structures were supported by high-resolution accurate mass spectrometry, and, for arsenolipid 444, confirmed by chemical synthesis. As-HC442 and As-HC440 were proposed, by analogy, to possess the same carbon skeletons as As-HC444, but with one or two double bonds in their structures, respectively. The position and geometry of the double bonds in arsenolipids As-HC440, As-HC442 and As-HC542 were not determined; they have been assigned by analogy to commonly occurring non-arsenic lipids in organisms^14^.

Considering these data, and comparison with normal hydrocarbons found in fish, molecular formulas for the compounds were postulated as C_25_H_50_AsO (molecular mass 440, two double bonds), C_25_H_52_AsO (molecular mass 442, one double bond), C_25_H_54_AsO (molecular mass 444, saturated) and C_33_H_56_AsO (molecular mass 542, seven double bonds). These molecular formulas were confirmed by accurate mass spectrometry (Table 1; Supplementary material Figs. S4–S7). In addition, the structure of one of the compounds (with molecular mass 444) was confirmed by chemical synthesis and comparison of the mass spectral and chromatographic properties of the natural and synthesised samples (Supplementary material, Figs. S8 & S9). Thus, the four new arsenolipids were assigned structures 8–11 (Fig. 2).

### The less-polar lipid fraction: (ii) Evidence for the presence of carboxylic acid esters

**Figure 3:**
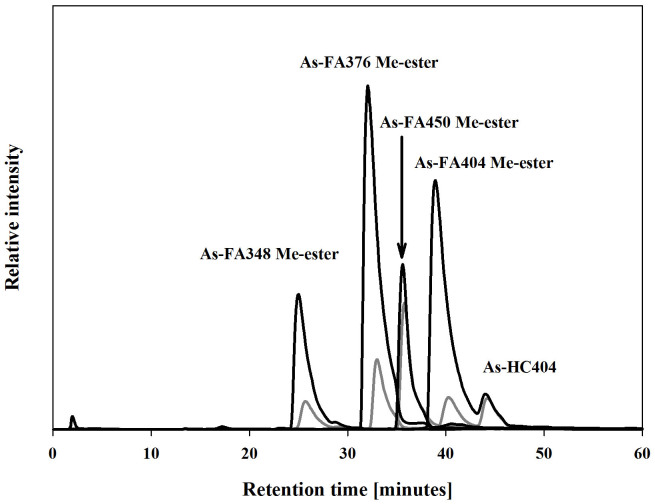
HPLC/ESI-MS chromatograms of methyl esters. The chromatograms show less polar arsenolipids (iso-propanol fraction) of blue whiting oil post-silica column (gray line), and the same sample spiked with four standard arsenic-containing fatty acid methyl esters at molecular mass 348, 376, 404 and 450 (black line), prepared by methylation of fatty acids isolated from cod liver oil^3^. HPLC conditions: Atlantis dC18 (150 × 1.0 mm, 5 μm) at 30°C and a mobile phase comprising a mixture of 10 mM NH_4_OAc pH 6.0 and ethanol at a flow rate of 100 μL min^−1^. The chromatography was performed with linear gradient elution: 0–60 min for 35%–95% ethanol. Selected ion monitoring was performed for [M+H]^+^ at *m/z* 349, 377, 451 and 405 with fragmentor voltage 150 V. Note: a second signal for *m/z* 405 ([M+H]^+^) belonged to compound **7** (As-HC404), an arsenic hydrocarbon in the isopropanol extract, which was not changed in the spiking experiment.

HPLC/ICPMS analysis of the isopropanol fraction pre- and post-silica column indicated that during the silica chromatography a considerable amount of the original arsenicals had been converted to more polar compounds (Supplementary material, Fig. S10). Analysis by HPLC/ESI-MS showed (Fig. 3) that four of the compounds were methyl esters of known arsenic-fatty acids^3^, three of which (**1**, **3** &, **4**) were identified in the blue whiting oil (see above). A fourth compound (mass 348) was the methyl ester of Me_2_As(O)(CH_2_)_12_COOH (As-FA334), which, although not detected in the blue whiting oil, had also been found in cod liver oil^3^.

**Figure 4:**
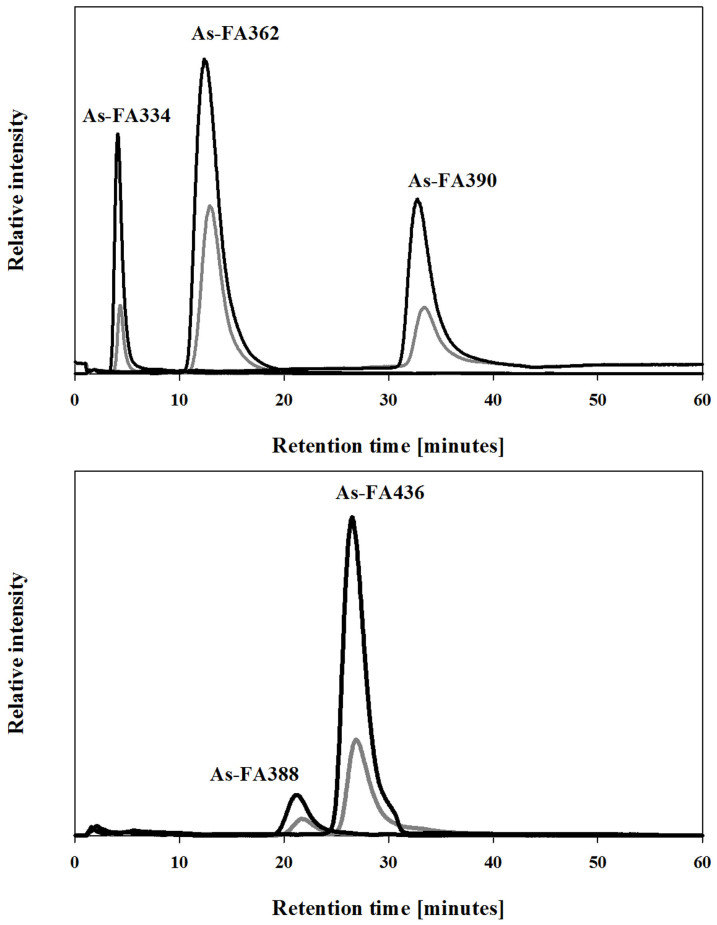
HPLC/ESI-MS chromatograms of hydrolysed lipid fractions. Water extract of the hydrolyzed iso-propanol fraction of blue-whiting oil (gray line) and the same sample spiked with standard arsenolipids previously isolated from cod liver oil^3^ (black line). HPLC conditions: Atlantis dC18 (150 × 1.0 mm, 5 μm) at 30°C and a mobile phase comprising a mixture of 10 mM NH_4_OAc pH 6.0 and ethanol at a flow rate of 100 μL min^−1^. The chromatography was performed with linear gradient elution: 0–60 min for 35%–50% ethanol. Selected ion monitoring was performed for [M+H]^+^ at *m/z* 335, 363, 391, 389 and 437 with fragmentor voltage 150 V.

The methyl esters were clearly artefacts from the silica/methanol chromatography. Bearing in mind that the original arsenicals were less polar than the methyl ester artefacts, the likely explanation was that transesterification of the original arsenic compounds had taken place on the column. To support this view, we hydrolysed the less polar lipid fraction, containing the purported fatty acid esters, and showed formation of five known As fatty acids (Fig. 4).

### In-situ on-column thiolation as a clean-up step for arsenolipids

The strong retention of arsenolipids on the silica column was presumed to be related to the basic nature of the Me_2_As(O)- group. Thus, modifying the arsenolipids after they have been applied to the column by converting the As = O group to an As = S group should reduce the compounds' attraction to silica, and their elution from the column might then be achieved with solvents less polar than methanol. The suitability of this approach, in terms of the stability of the compounds and recovery, was tested with the model arsenolipids, compound **5** (As-HC332) and the methyl ester of compound **1** (As-FA 362). The compounds were applied in diethyl ether/acetone whereupon they were retained, and then quantitatively recovered as the thio derivative by washing the column with H_2_S/acetone.

The method was then applied to the polar lipid fraction (aq. MeOH) of blue whiting oil. Most of the impurities (>80% by mass), but only a trace of arsenic (<2% As), were eluted with diethyl ether/acetone. The arsenic compounds were then eluted with H_2_S/acetone yielding a fraction greatly enriched in arsenic which was amenable to HPLC/ESI-MS analysis (Supplementary Fig. S11). In this way, four major arsenolipids (**1**, **3**, **4**, and **7**) were identified as their thio analogues, confirming our earlier analysis of the polar lipid fraction.

## Discussion

In addition to reporting seven known compounds in blue whiting oil, this study has added four new compounds to the growing list of naturally occurring arsenolipids, and provided evidence for the presence of conjugates of arsenic-fatty acids. We estimate that these conjugated fatty acids constitute about 25% of the total arsenic content in the blue whiting oil (2.16 μg As g^−1^). The various arsenolipids found in marine organisms likely reflect the natural abundance of the non-arsenic lipids, e.g. major arsenic lipids, by abundance, are based on palmitic acid and its derivatives, which are major compounds in marine oils. In that event, the arsenolipids might be a measure of the fidelity of biosynthetic processes designed for essential compounds. There is no evidence to suggest that this “wrong direction” or even the accumulation of arsenic-containing lipids in the organisms is detrimental.

The possibility remains, however, that the arsenolipids might serve some purpose in the organisms. Further investigations of the biological implications of arsenolipids are dependent on analytical methods to identify and quantify the various compounds^15^,^16^,^17^,^18^. One of the crucial analytical steps is a clean-up procedure that can capture the arsenolipids and separate them from the vast excess of normal lipids in the samples. Our attempts to clean-up the samples with silica chromatography have been informative in two ways.

First, the arsenolipids show a great affinity for silica, presumably because almost all of them contain the basic Me_2_As(O)- group. Thus, a very efficient separation from normal lipids can be achieved by applying lipid extracts to a silica column and washing the normal lipids off with solvents of low to moderate polarity. However, the arsenolipid-silica affinity is so strong that large volumes of highly polar solvents (e.g. MeOH) are then required to elute the arsenolipids. This problem is solved by washing the column with H_2_S/acetone to convert the oxo-arsenolipids to their less polar thio analogues, which are then easily eluted from the column. When the method was applied to blue whiting oil, the major arsenolipids were recovered in good yield with a large improvement in purity. However, the suitability of this silica/thiolation procedure to other more complex arsenolipids is yet to be demonstrated.

**Figure 5:**
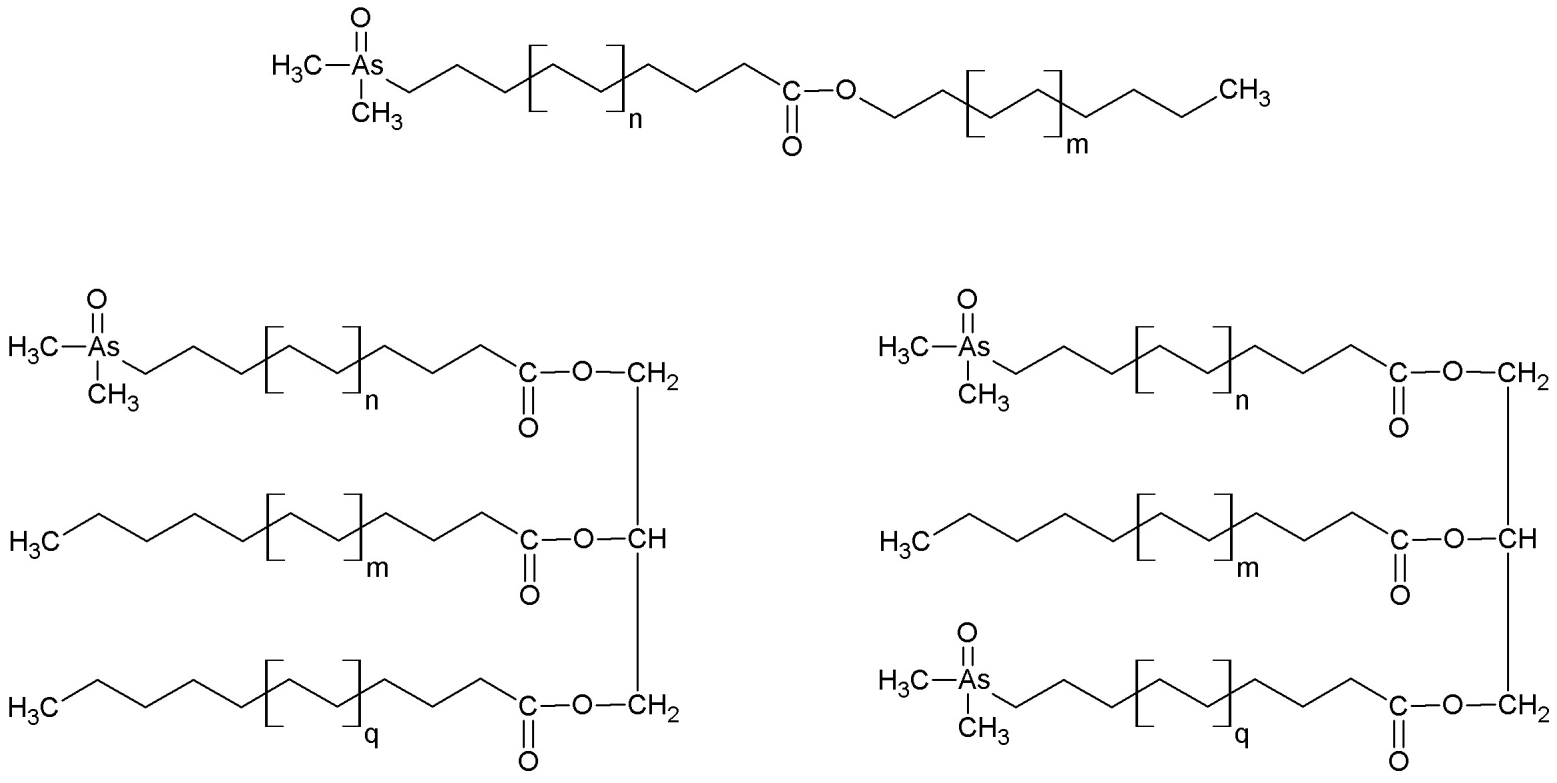
Some postulated structures for less polar arsenolipids. Such derivatives of wax esters/triglycerides in the less polar fraction (extracted with aqueous isopropanol) of blue whiting oil could give rise to the mixture of arsenic-fatty acids observed following hydrolysis.

Second, the use of silica/methanol converted a large amount of less-polar arsenolipids to several carboxylic acid methyl esters. These artefacts from the clean-up procedure could not have arisen from methylation of fatty acids in the sample because those compounds were not present in the less polar fraction. Additionally, when a standard arsenic fatty acid was subjected to silica/methanol chromatography, it did not form the corresponding methyl ester. The most likely explanation is that transesterification of the original arsenic compounds had taken place on the column. The quantitative transesterification of ester lipids to methyl esters using silica gel as a catalyst and methanol has been reported^19^. Furthermore, when a freshly prepared non-acidic fraction of the less polar blue whiting lipids was prepared and base-hydrolysed, five arsenic-fatty acids were produced (Fig. 4). These results confirmed that conjugated arsenic-containing fatty acids, possibly wax esters or more likely triglycerides, are present in the less polar lipid fraction of blue whiting oil (Fig. 5).

Full structural characterisation of these new arsenolipids might provide clues as to a possible role for these compounds in organisms. For example, the presence of more than one arsenic-containing fatty acid in a single triglyceride would indicate directed and controlled biosynthesis of the compounds; because the arsenic fatty acids are much less abundant than the normal fatty acids a purely random doubling-up of arsenic fatty acids would be most improbable. Should directed and controlled biosynthesis of arsenolipids be found, a biological role for these compounds would then appear more likely. We continue our investigations on the analytical and chemical aspects relevant to delineating a possible biological role for arsenic.

## Methods

The oil from the blue whiting, *Micromesistius poutassou*, was a crude unrefined oil obtained by heating and pressing the whole fish. It was a product from the Republic of Iceland.

### Determination of total arsenic and arsenic species, and accurate masses

Total arsenic was determined by Inductively Coupled Plasma Mass Spectrometry (ICPMS) after mineralization of the samples with microwave-assisted acid digestion. Specified portions of samples, extracted solutions (fractions), or certified reference materials were weighed into quartz tubes (12 mL) and concentrated nitric acid (2–4 mL) was added. The quartz tubes were then closed with Teflon^®^ caps and placed into the sample rack of the autoclave (ultraCLAVE III, EMLS, Leutkirch, Germany). The autoclave was loaded with argon to a pressure of 40 bar (4 × 10^6^ Pa), the samples were heated to 260°C, and this temperature was maintained for 40 min. The digests were then cooled, and diluted (20–50 mL, depending on the sample) with water in 50 mL polypropylene tubes. Arsenic concentrations in these digest solutions were determined by external calibration against arsenic standards (from CPI International, Amsterdam, Netherlands), with an Agilent ICPMS 7500 ce (Agilent Technologies, Waldbronn, Germany) equipped with an ISIS (Integrated Sample Introduction System), an ASX-500 auto-sampler and a Microflow® PFA-100 nebulizer. Measurements were made in collision cell mode with He as collision gas at 4 mL min^−1^, using ^72^Ge and ^115^In as internal standards (to normalise arsenic signals). The accuracy of total arsenic determinations was checked by analysing certified reference material Dorm-2 (National Research Council of Canada, Ottawa, Canada; certified arsenic concentration 18.0 ± 1.1 μg g^−1^); the overall measured mean arsenic concentration during the course of this study was 18.3 ± 1.0 μg g^−1^ (mean ± SD, n = 9). All samples (original oil, extract solutions and fractions) were analysed in triplicate; the values are expressed as mean [As] ± SD (n = 3).

Arsenic species were determined by reversed-phase HPLC coupled to either an ICPMS or an electrospray MS (both Agilent single quadrupole instruments); conditions are recorded in the legend to the figures containing the chromatograms. High resolution accurate mass measurements were performed on a LTQ-Velos mass spectrometer (Thermo Fisher Scientific), equipped with a nano electrospray source. Dried samples were dissolved to a concentration of approximately 10 μM in ethanol containing 1% formic acid and sprayed from metal-coated borosilicate emitters (Proxeon, Odense). Instrument resolution was set to 60,000 (Full Width at Half Maximum, FWHM), Spray voltage 1000 V, Capillary temperature: 200°C. The exact MS measurements was obtained using three lock masses (389.2510, 447.2929, 505.3347 sodium ions) from added polyethylene glycol.

### Fractionation of polar arsenolipids

A flow chart outlining the fractionation steps is provided in the Supplementary material (Fig. S2). Blue whiting oil (200 g with 2.16 μg As g^−1^ representing a total of 432 μg As) was weighed into a 1000 mL separatory funnel, dissolved in hexane (500 mL) and extracted with aqueous methanol (1 + 9, v/v; 300 mL) which was then evaporated to yield an oil (1.0 g; 103 μg As). This procedure was repeated with aqueous methanol (1 + 9, v/v; 300 mL yielding 45 μg As in 1 g oil), and aqueous ethanol (3 + 17 v/v; 300 mL yielding 37 μg As in 1.2 oil). These two fractions were not further investigated because HPLC/ICPMS analysis revealed that they contained the same arsenic compounds as the first aqueous methanol extract but at considerably lower concentrations. Preparative anion-exchange chromatography was performed on the first aqueous methanol extract with DEAE Sephadex A-25 column (240 mm × 26 mm) and methanol/chloroform/aqueous sodium acetate (60 + 30 + 8, v/v/v) as previously described^3^. Most of the arsenic was eluted near the void volume of the column (the non-acidic fraction, 145 mg, 67 μg As), whereas the acidic arsenic (170 mg including acetate buffer, 14 μg As) eluted between 1150–1250 mL.

### Fractionation of less polar arsenolipids

The hexane layer from the above partitioning was extracted with aqueous isopropanol (3 + 17 v/v; 2 × 300 mL) and the alcohol layer was evaporated to yield an oil (9.5 g, 130 μg As). The extracted hexane layer (non-polar lipids, 173 g, 143 μg As) was not further examined. HPLC/ICPMS analysis of the isopropanol layer showed that most of the arsenic (>95%) was present as less polar lipids with only traces of the polar and non-polar lipids.

### Treatment of less polar arsenolipids with silica column/methanol

To a silica column (40 g; 20 × 300 mm, equilibrated in acetone) was applied a portion of the aqueous isopropanol fraction (3.4 g, 47 μg) in acetone, and the column was eluted with acetone (520 mL) to yield an oil (3.1 g, 0.7 μg As) and then methanol (1650 mL) to yield a solid/oil (170 mg, 39 μg As). A portion of the methanol fraction was used for direct analysis by HPLC/ESI-MS and HPLC/ICPMS. A further portion was purified for high resolution mass spectrometry by HPLC (Atlantis C18 column; 150 × 4.6 mm, 5 μm particles) and a mobile phase comprising a mixture of 92% ethanol-buffer (100 mM NH_4_OAc, pH 5.0) (85 + 15, v/v) and 8% chloroform at a flow rate of 0.5 mL min^−1^ (isocratic elution). The effluent of the HPLC was divided using a splitter (Active splitter, G1968D, Agilent, USA) which directed 1/5 of the flow to the ESI-MS, and the remainder to a fraction collector which collected 1.5 mL volumes. ESI-MS was operated in positive ion mode with fragmentor voltage of 400 V at *m/z* 91 to detect arsenic as [AsO]^+^ and also with fragmentor voltage of 150 V to determine the protonated molecular ion (*m/z* [M+H]^+^) from individual expected arsenolipids.

Synthesized arsenic fatty acid **1** (As-FA362, ca 100 μg)^20^ similarly exposed to silica/methanol As in 1 mL methanol) showed a recovery of >85% and there was no detectable amounts of its methyl ester (monitored by ESI-MS).

### Hydrolysis of less polar arsenolipids with ethanolic KOH

A portion (3.8 g, 53 μg As) of the aqueous isopropanol fraction was subjected to anion-exchange chromatography with DEAE Sephadex A-25 as described above. Most of the arsenic (>95%) was eluted near the void volume of the column to give the non-acidic fraction (oil, 1.6 g). A late eluting fraction represented small amounts of acidic arsenic (ca 600–700 mL; 1.8 μg As). A portion (430 mg, 15 μg As) of the non-acidic fraction was hydrolyzed with ethanolic potassium hydroxide solution (0.5 M, 12 mL) at 70°C for two hours in a closed flask. The ethanol was removed in vacuo and then water (20 mL) and diethyl ether (50 mL) were added to the residue. The solution was acidified (HCl) to pH 3, the mixture was shaken, and the aqueous layer was separated, neutralized with 2 M potassium hydroxide, and evaporated in vacuo to dryness to leave a residue containing 8 μg As, which was analyzed by HPLC/ESI-MS. When this procedure was repeated on the synthesized methyl ester of arsenolipid **1** (As-FA362, 20 μg As), the free arsenic fatty acid was formed (>85% yield) and no methyl ester remained.

### In-situ on-column thiolation as a clean-up step for arsenolipids

Silica gel (10 g) was equilibrated in diethyl ether, and the slurry was poured into a column (10 mm × 100 mm). A portion of the aq. methanol fraction (polar lipids, 1.0 g, 65 μg As) was dissolved in diethyl ether and loaded onto the column. The column was initially eluted with diethyl ether (80 mL), followed by acetone (40 mL), and then H_2_S in acetone (40 mL). The diethyl ether and acetone fractions were concentrated to yield oils (0.63 g, 0.6 μg As, and 0.13 mg, 0.2 μg As, respectively). Most of the arsenic was eluted in the H_2_S/acetone fraction, which was concentrated to yield an oily residue (106 mg, 51 μg As) which was analysed by HPLC/ESI-MS.

### Synthesis of compound 11 (As-HC444; 1-dimethylarsinoyltricosane

1-Tricosanol was converted to the triflate using trifluoromethanesulfonic acid anhydride in dichloromethane, and treated with sodium dimethylarsenide in THF at −78°C. The resulting arsine was oxidized with H_2_O_2_ and the product recrystallised from acetone to yield 1-dimethylarsinoyltricosane as a white powder which was characterized by high-resolution mass spectrometry: *m/z* calculated for C_25_H_53_AsO ([M+H]^+^) 445.3390, found 445.3389 (Δm/m = < 0.3 ppm). This product was identical with that obtained by a different, higher yielding, synthetic route, and recently fully characterised^20^.

## Author Contributions

M.S.T. performed the experimental work; G.R. performed HPLC/MS measurements; J.S.E. advised on and contributed to the purification of compounds; K.B.J. performed accurate mass analyses; and K.A.F. planned the study and wrote the manuscript. All authors read and approved the manuscript.

## Supplementary Material

Supplementary Information 141003 SREP-14-05959 - Revised Supp material

## Figures and Tables

**Table 1:** Accurate mass data for the four new arsenolipids in blue-whiting oil

Arsenolipid	Molecular formula	[M+H]^+^ Calc.	[M+H]^+^ measured	(Δm/m, ppm)
As-HC440	C_25_H_49_AsO	441.3077	441.3075	0.5
As-HC442	C_25_H_51_AsO	443.3234	443.3235	<0.3
As-HC444	C_25_H_53_AsO	445.3390	445.3385	1.1
As-HC542	C_33_H_55_AsO	543.3547	543.3545	0.4
