# Supplementary material for: Arsenolipids in oil from blue whiting Micromesistius poutassou – evidence for arsenic-containing esters
Source: Sci Rep. 2014 Dec 15;4:7492. doi: 10.1038/srep07492 (PMC4265782; doi:10.1038/srep07492)
Supplement: Supplementary Information — 141003 SREP-14-05959 - Revised Supp material [file srep07492-s1.doc]

**SUPPLEMENTARY MATERIAL FOR**

**Arsenolipids in oil from blue whiting *Micromesistius poutassou* – evidence for arsenic-containing esters**

Mojtaba S. Taleshi^1,2^, Georg Raber^1^, John S. Edmonds^1^, Kenneth B. Jensen^1^, and Kevin A. Francesconi^1^*

^1^Institute of Chemistry-Analytical Chemistry, University of Graz, Universitaetsplatz 1, 8010 Graz, Austria

^2^Department of Marine Chemistry, Faculty of Marine Science, University of Mazandaran, Babolsar, Iran

*Corresponding author

[kevin.francesconi@uni-graz.at](mailto:kevin.francesconi@uni-graz.at)

The following Supplementary material accompanies this paper:

Figure S1. Classification of arsenolipids.

Figure S2. Flow diagram for fractionation of arsenolipids in blue whiting oil.

Figure S3. HPLC/ESI-MS chromatogram of arsenolipids in the isopropanol extract of blue-whiting oil, post-silica column.

Figure S4. High resolution accurate mass spectrum for As-HC440 in blue-whiting oil.

Figure S5. High resolution accurate mass spectrum for As-HC442 from blue-whiting oil.

Figure S6. High resolution accurate mass spectrum for As-HC444 in blue-whiting oil.

Figure S7. High resolution accurate mass spectrum for As-HC542 in blue-whiting oil.

Figure S8. HPLC/ESI chromatograms of As-HC444).

Figure S9. High resolution mass spectrum for synthesized As-HC 444.

Figure S10. HPLC/ICPMS chromatogram of the less polar arsenolipids (iso-propanol fraction) of the blue-whiting oil.

Figure S11. HPLC/ESI-MS chromatograms of thio analogues of arsenolipids

**Non-polar**

**(Unknown structures)**

**Arsenolipids**

**Polar**

**(As fatty acids**

**+ smaller As hydrocarbons)**

**Less polar**

**(As hydrocarbons**

**+ unknowns)**

**Figure S1.** **Classification of arsenolipids.**

On the basis of their extraction by solvent partitioning, and their retention on reversed-phase HPLC, arsenolipids in fish oils can be categorized into three broad polarity groups: polar (mainly arsenic-containing fatty acids), less-polar (mainly arsenic-containing hydrocarbons), and non-polar (containing unknown arsenolipids). In this example of crude blue whiting oil, HPLC conditions were: Atlantis dC18 (150🞨1.0 mm, 5 µm) at 30 ^o^C and a mobile phase comprising a mixture of 10 mM NH_4_OAc pH 6.0 and ethanol at a flow rate of 100 µL min^-1^. The chromatography was performed with linear gradient elution: 0−60 min for 35%−95% ethanol).

**Blue-Whiting Oil**

2.16 µg As/g

200.7 g, 434 ± 2 µg As

**MeOH (1) fraction**

1.0 g, 103 ± 6 µg As

**Hexane (1) fraction**

199 g, 333 ± 7 µg As

**MeOH (2) fraction**

1.0 g, 45 ± 2 µg As

**Hexane (2) fraction**

195 g, 297 ± 7 µg As

**EtOH fraction**

1.2 g, 37 ± 1 µg As

**Hexane (3) fraction**

190 g, 260 ± 4 µg As

**IPA fraction**

9.5 g, 130 ± 2 µg As

**Hexane (4) fraction**

173 g, 143 ± 9 µg As

MeOH_aq_ (300 mL)

EtOH_aq_ (300 mL)

Isopropanol_aq_ (IPA) (2🞨300 mL)

**Hexane (3) fraction**

184 g, 252 ± 3 µg As

DEAE Sephadex

**Acidic fraction**

170 mg, 14 ± 0.3 µg As

Basic/neutral **fraction**

145 mg, 67 ± 3 µg As

Dissolved in hexane (500 mL)

Extracted with MeOH_aq_ (300 mL)

**Figure S2. Flow diagram for fractionation of arsenolipids in blue whiting oil.**

**Figure S3. HPLC/ESI-MS chromatogram of arsenolipids in the isopropanol extract of blue-whiting oil, post-silica column.** a) Measured at *m/z* 91 with fragmentor voltage 400 V and b) Performed in scan mode (*m/z* 100-1000) with fragmentor voltage 150 V and the masses were extracted from scan mode. HPLC conditions: Atlantis dC18 (150 × 4.6 mm, 5 µm) at 30 ^o^C and a mobile phase comprising a mixture of 92% ethanol-buffer (100 mM NH_4_OAc, pH 5.0) (85+15, v/v) and 8% chloroform at a flow rate of 0.5 mL min^-1^ (isocratic elution).

Arsenolipid 440

**Figure S4. High resolution accurate mass spectrum for As-HC440 in blue-whiting oil.** Molecular formula C_25_H_49_AsO: calculated for [M+H]^+^ 441.3077; found 441.3075; Δm/m = 0.5 ppm.

Arsenolipid 442

**Figure S5. High resolution accurate mass spectrum for As-HC442 from blue-whiting oil.** Molecular formula C_25_H_51_AsO: calculated for [M+H]^+^ 443.3234; found 443.3235; Δm/m < 0.3 ppm.

Arsenolipid 444

**Figure S6. High resolution accurate mass spectrum for As-HC444 in blue-whiting oil.** Molecular formula C_25_H_53_AsO: calculated for [M+H]^+^ 445.3390; found 445.3385; Δm/m = 1.1 ppm.

Arsenolipid 542

**Figure S7. High resolution accurate mass spectrum for As-HC542 in blue-whiting oil.** Molecular formula C_33_H_55_AsO: calculated for [M+H]^+^ 543.3547; found 543.3545; Δm/m = 0.4 ppm.

**Figure S8. High resolution mass spectrum for synthesized As-HC 444.** Molecular formula C_25_H_53_AsO: calculated for [M+H]^+^ 445.3390; found 445.3389; Δm/m < 0.3 ppm.


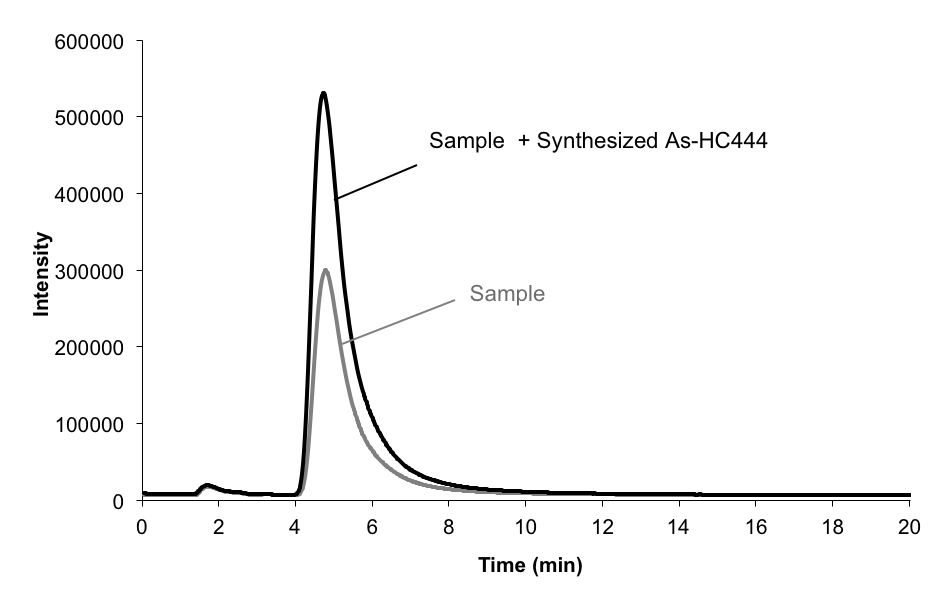


**Figure S9. HPLC/ESI-MS chromatograms of As-HC444).** Purified As-HC444 from the isopropanol-fraction of blue-whiting oil (gray line) and the same sample spiked with synthesized As-HC444 (solid line). HPLC conditions: Atlantis dC18 (150 × 1.0 mm, 5 µm) at 30 ^o^C and a mixture of 85% of ethanol-buffer (100 mM NH_4_OAc, pH 5.0) (9+1) and 15% chloroform (isocratic elution) at a flow rate of 100 µL min^-1^. Selected ion monitoring was performed for [M+H]^+^ at *m/z* 445 with fragmentor voltage 150 V.


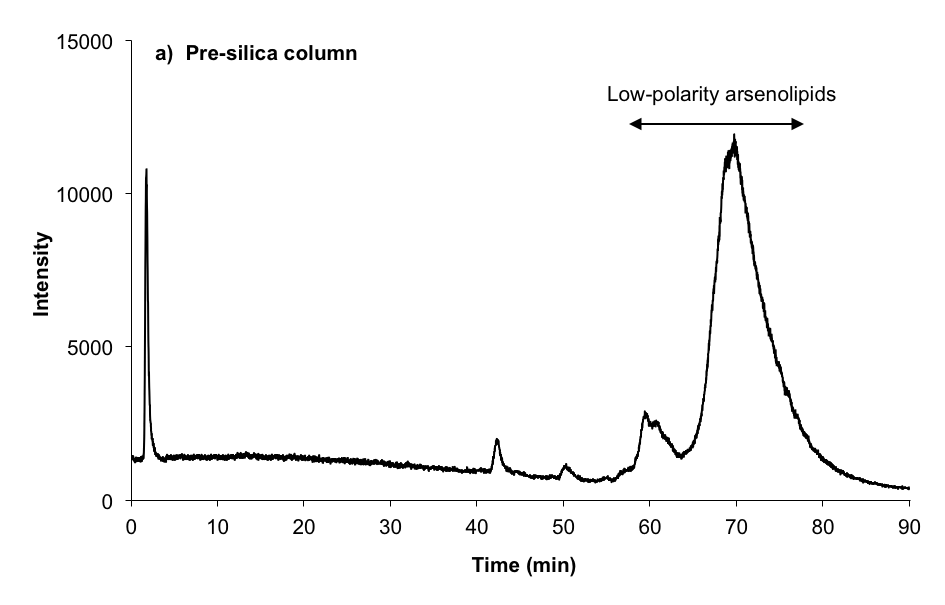

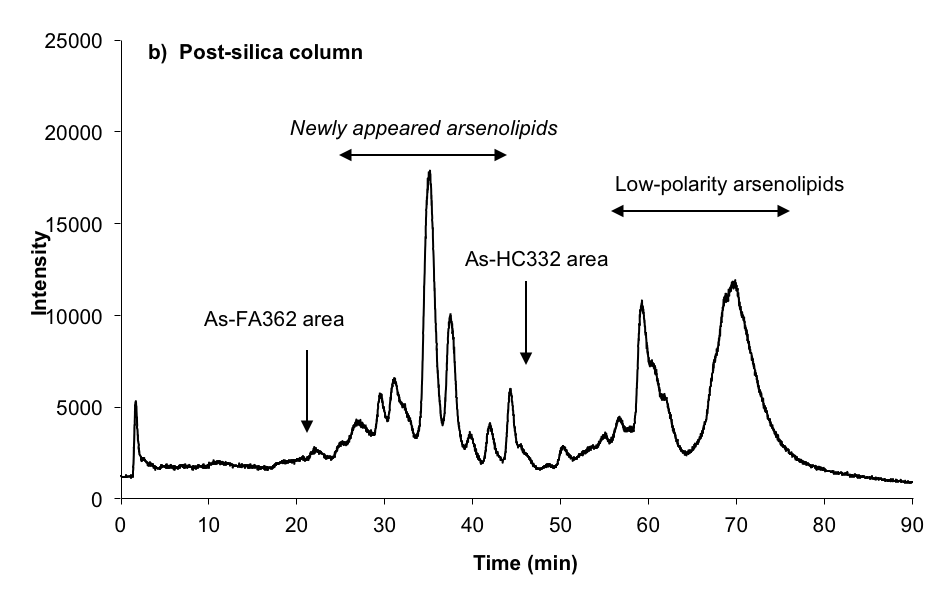


**Figure S10. HPLC/ICPMS chromatogram of the less polar arsenolipids (iso-propanol fraction) of the blue-whiting oil.** a) Pre-silica column and b) post-silica column. The newly appeared arsenolipids showed peaks between those of arsenic-containing fatty acids (e.g. As-FA362) and arsenic-containing hydrocarbons (e.g. As-HC332). HPLC conditions, for both a) and b) were: Atlantis dC18 (150🞨1.0 mm, 5 µm) at 30 ^o^C and a mobile phase comprising a mixture of 10 mM NH_4_OAc pH 6.0 and ethanol at a flow rate of 100 µL min^-1^. The chromatography was performed with linear gradient elution: 0−60 min for 35%−95% ethanol.


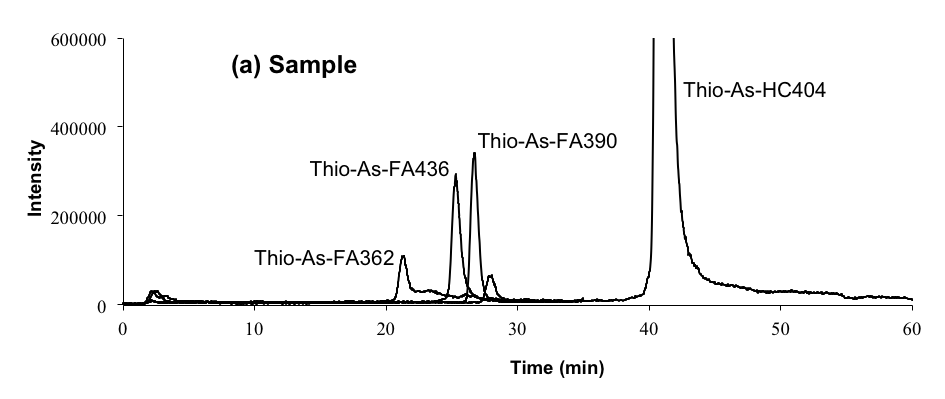


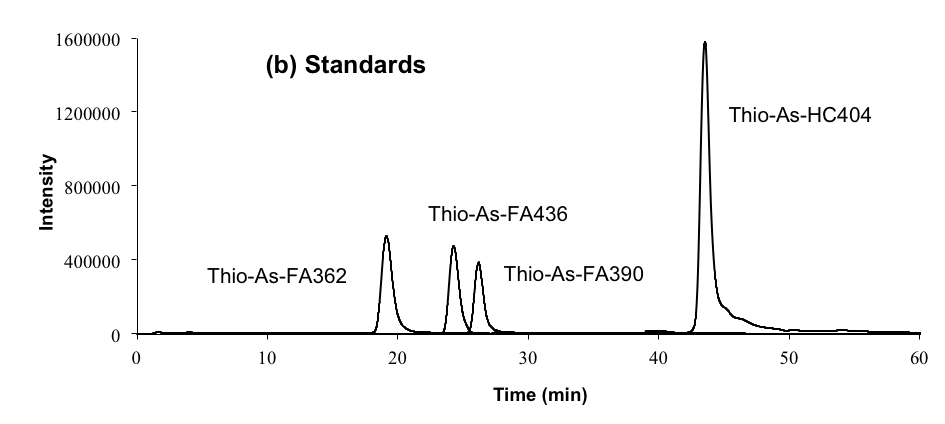


**Figure S11.**  **HPLC/ESI-MS chromatograms of thio analogues of arsenolipids**. (a) polar fraction of blue whiting oil post-silica column after elution with H_2_S/acetone; and (b) thio-arsenolipid standards prepared by bubbling of H_2_S gas into standard solutions of oxo arsenolipids As-FA362, As-FA436, As-FA390, and As-HC404. HPLC conditions: Atlantis dC18 (150 × 1.0 mm, 5 µm) at 30 ^o^C and a mobile phase comprising a mixture of 10 mM NH_4_OAc (pH 6.0) and ethanol at a flow rate of 100 µL min^-1^. The chromatography was performed with linear gradient elution: 0−60 min with 35%−95% ethanol. Selected ion monitoring was performed for [M+H]^+^ at *m/z* 379, 453, 407, 421 and 377 with fragmentor voltage 150 V. The slight differences in retention times are attributed to small changes in column performance between the HPLC runs.
